# Comparative analysis of e-cigarette prevalence and influencing factors among adolescents in Jiangsu Province, China

**DOI:** 10.3389/fpubh.2023.1221334

**Published:** 2023-12-01

**Authors:** Jiannan Fan, Tao Mao, Shiqi Zhen, Yan Xu, Chen Qu

**Affiliations:** ^1^Institute of Health Education, Jiangsu Provincial Center for Disease Control and Prevention, Nanjing, Jiangsu, China; ^2^Key Laboratory of Environmental Medicine Engineering, Ministry of Education, School of Public Health, Southeast University, Nanjing, Jiangsu, China

**Keywords:** e-cigarette, tobacco experimentation, adolescents, association, health education

## Abstract

**Objective:**

The objectives of this study were to investigate electronic cigarette (e-cigarette) and cigarette use in Jiangsu Province, China, by analyzing the two-year trends of e-cigarette using and to explore the factors influencing the experimentation and use of e-cigarettes.

**Methods:**

We conducted a cross-sectional study following the standard methodology of the Global Youth Tobacco Survey in 2019 and 2021. A three-stage cluster sampling design was applied. Eighty-two schools in 14 districts (counties) in Jiangsu Province were surveyed. All computations were performed using the SPSS 21.0 complex samples procedure. Multivariate logistic regression was used to explore the factors influencing e-cigarette experimentation and use.

**Results:**

A total of 12,410 and 12,880 students were surveyed in 2019 and 2021, respectively. E-cigarette experimentation increased from 9.34% in 2019 to 13.07% in 2021 (*P* < 0.001). E-cigarette use increased from 2.23% in 2019 to 3.74% in 2021 (*P* < 0.001). The main factors associated with e-cigarette use were cigarette experimentation (OR = 2.700, *P* < 0.001); male gender (OR = 1.416, *P* = 0.011); junior high school students (OR = 1.551, *P* = 0.005) and vocational high school students (OR = 1.644, *P* = 0.001); more pocket money per week (OR_1_ = 1.214, *P* = 0.187; OR_2_ = 1.686, *P* = 0.001); exposure to second-hand smoke (SHS) at home (OR = 1.239, *P* < 0.001); exposure to e-cigarette advertising (OR = 1.855, *P* < 0.001); believe SHS is harmful (OR = 0.933, *P* = 0.026); closest friends smoking (OR = 2.501, *P* < 0.001); believe smoking makes youth look more attractive (OR_1_ = 1.469, *P* = 0.040; OR_2_ = 1.305, *P* = 0.049); believe tobacco helps youth feel more comfortable in social situations (OR_1_ = 2.161, *P* < 0.001; OR_2_ = 1.635, *P* = 0.001); will use an e-cigarette product if offered by best friends (OR = 1.322, *P* < 0.001); intend to use an e-cigarette product in the next 12 months (OR = 1.486, *P* < 0.001).

**Conclusion:**

E-cigarette use among adolescents has been on the rise in recent years. E-cigarette use is associated with past cigarette use and a strong desire to smoke. It is crucial to take health education and tobacco control efforts to reduce adolescents' e-cigarette use rate.

## 1 Introduction

Electronic cigarettes (e-cigarettes) are gradually becoming a popular tobacco product with the development of the economy. They are the most popular tobacco products among high school students in the United States (1), and 11.3% of high school students and 2.8% of middle school students were reported to be using e-cigarettes in 2021 (2). Smoking among adolescents is both a psychological and social behavior that is influenced by a variety of factors (3). Family, school, friends, tobacco marketing, policies and regulations are environmental factors that influence youth smoking behavior (4). Adolescents are good at imitation, lack proper knowledge of the dangers of tobacco, and believe that smoking makes them more popular socially, thus making it easy for them to develop smoking behaviors during adolescence. Many adult smokers develop their smoking habits as adolescents (5).

Smoking is a leading cause of premature mortality and morbidity. Acute effects of smoking have been reported in the pulmonary, cardiovascular, and immune systems. E-cigarettes and their delivered toxicants appear harmful to multiple organ systems, increasing the burden of symptoms due to coronavirus disease 2019 (COVID-19) and may lead to severe health consequences (6–8). E-cigarettes also contain addictive nicotine (9).

E-cigarette markets are gradually expanding, and the promotion channels have become diverse (10). Many companies have launched their brands of e-cigarettes in recent decades, and sales of e-cigarettes are catching up with traditional cigarettes (11). E-cigarette advertisements are everywhere on the street. E-cigarettes are now more readily available to adolescents (12). E-cigarette markets even launch candy fruit flavors specifically marketed to adolescents and children (13, 14). Both current and future smoking risks are highly correlated with e-cigarette use (15). Legislation prohibiting smoking in public places and banning tobacco advertising and promotion can effectively reduce the prevalence of smoking among adolescents (16).

Despite the growing popularity and prevalence of e-cigarettes worldwide, the concrete data and research on e-cigarettes are insufficient. Following the standard methodology of the Global Youth Tobacco Survey (GYTS), Jiangsu Province conducted two tobacco epidemic surveillance efforts in 2019 and 2021. It is a general epidemiological investigation. Jiangsu Province is located in the eastern coastal region of China, and its gross domestic product is the second highest in China. The survey conducted in Jiangsu Province can thus provide a good reference of the situation in reference China. GYTS is a school-based survey whose purpose is to compare and analyze the changes in e-cigarette and cigarette epidemic characteristics in recent years. It captures the influences of tobacco use among adolescents and provides a scientific basis for youth tobacco control efforts (2, 17). This survey is representative of the provincial level.

E-cigarette vaping status among Chinese adults increased from 1.3% in 2015 to 1.6% in 2018, showing an increasing trend year by year (18). The smoking rate of adolescents has a strong correlation with that of future adults. An adolescent who had experimented with smoking is likely to develop into a smoker in the future (16). Most adult smokers (about 90%) started smoking even before the age of 18 years (2). Adolescents' tobacco control efforts are necessary to control adult smoking and hence reduce adult smoking rates. Therefore, we explored the factors influencing smoking experimentation among adolescents. We investigated adolescents' cigarette and e-cigarette experimentation and use rates in 2019 and 2021, providing baseline data on cigarette and e-cigarette smoking. We also compared the e-cigarette vaping status with different characteristics and explored the factors influencing adolescents' experimentation and use of e-cigarettes.

## 2 Methods

### 2.1 Study population and district

This study was conducted in Jiangsu in 2019 and 2021. The survey included middle school, academic high school, and vocational high school students. The survey design, sampling method, and questionnaire adopted in 2019 and 2021 were the same. The sample number and characteristics are summarized in Table 1. “Unweighted” refers to the final number of students surveyed. “Number” is the number of people these students can represent. A total of 12,410 students were surveyed in 2019, which could represent 3,870,045 students in the whole Jiangsu province. Furthermore, 12,880 students were surveyed in 2021, which could represent 4,307,601 students. Boys accounted for 53.46% of this total, while girls accounted for 46.54% in 2019. Boys constituted 53.96% of the total, and girls constituted 46.04% of the total in 2021. Students in urban areas accounted for 50.41% of this total, and those in rural areas accounted for 49.59% in 2019. Students in urban areas accounted for 51.08%, and rural areas accounted for 48.92% of the total in 2021. Students in junior high school, academic high school, and vocational high school accounted for 58.84%, 25.56%, and 15.61% of the total, respectively, in 2019. Finally, students in junior high school, academic high school, and vocational high school accounted for 59.15%, 26.70%, and 14.15% of the total, respectively, in 2021.

**Table 1:** Characteristics of Jiangsu adolescents.

		**2019**			**2021**		
		**Weighted proportion% (95% CI)**	**Number**	**Unweighted**	**Weighted proportion% (95% CI)**	**Number**	**Unweighted**
Overall		100	3,870,045	12,410	100	4,307,601	12,880
Gender	Boys	53.46 (51.19–55.70)	2,068,750	6,919	53.96 (52.05–55.86)	2,324,417	7,177
	Girls	46.54 (44.30–48.81)	1,801,295	5,491	46.04 (44.14–47.95)	1,983,184	5,703
Residence	Urban	50.41 (40.98–59.80)	1,950,789	6,909	51.08 (40.93–61.15)	2,200,487	7,280
	Rural	49.59 (40.20–59.02)	1,919,256	5,501	48.92 (38.85–59.07)	2,107,114	5,600
School type	Junior high school	58.84 (46.93–69.79)	2,277,035	6,352	59.15 (47.54–69.82)	2,547,835	6,380
	Grade 1	20.96 (16.99–25.58)	811,273	2,168	20.47 (16.84–24.64)	881,697	2,161
	Grade 2	19.74 (15.99–24.11)	763,864	2,108	20.08 (16.40–24.36)	865,116	2,152
	Grade 3	18.14 (14.86–21.95)	701,898	2,076	18.60 (15.27–22.45)	801,022	2,067
	Academic high school	25.56 (17.88–35.12)	989,070	4,303	26.70 (18.79–36.45)	1,150,243	4,576
	Grade 1	9.19 (6.50–12.85)	355,843	1,519	9.82 (6.99–13.62)	422,860	1,500
	Grade 2	8.26 (5.86–11.53)	319,716	1,401	8.89 (6.26–12.47)	382,886	1,580
	Grade 3	8.10 (5.70–11.39)	313,511	1,383	8.00 (5.73–11.05)	344,497	1,496
	Vocational high school	15.61 (8.30–27.42)	603,940	1,755	14.15 (7.49–25.12)	609,523	1,924
	Grade 1	5.17 (2.83–9.28)	200,257	645	5.13 (2.69–9.57)	220,985	641
	Grade 2	5.37 (2.88–9.80)	207,751	589	4.77 (2.48–8.98)	205,358	605
	Grade 3	5.06 (2.65–9.45)	195,932	521	4.25 (2.33–7.63)	183,180	678

### 2.2 Sampling method

Representative students were sampled using a three-stage cluster sampling design. In the first stage, 14 monitoring districts (counties) were randomly selected in the province using probability proportionate to size (PPS) sampling. In the second stage, three junior high schools, two academic high schools, and one vocational high school were selected via PPS sampling in each monitoring district (county). Schools used for sampling included all public and private schools in the region, and classes with less than 40 people were excluded. Finally, 82 schools were selected. In the third stage, one class from each grade in each school was randomly selected, and all students in the class present on that day participated.

### 2.3 Measure

The GYTS China Project was conducted. The questionnaire inquired about an individual's key social-demographic characteristics (e.g., age, gender, school type, tobacco smoking, and pocket money each week), predisposing factors of tobacco use (e.g., cognition concerning dangers of tobacco use, acceptance of tobacco use, and exposure to smoking by parents and friends), enabling and reinforcing factors (e.g., second-hand smoke [SHS] exposure and tobacco advertising exposure), smoking dependence and susceptibility. The questionnaire showed suitability by the reliability and validity analysis (19).

### 2.4 Definitions of various indicators

The different indicators used in the survey are described as follows:

*Smoking experimentation* was deduced by enquiring if the respondent ever tried or experimented with cigarette (e-cigarette) smoking, even one or two puffs.

*Current smoker* was inferred by asking about the number of days the respondent smoked a cigarette (e-cigarette) in the past 30 days. If the response was 1 day or more, the respondent was identified as a current smoker.

*SHS exposure* was found by asking if the respondent saw someone smoke in a place (home, indoors, outdoors, public transport, school) in the past seven days.

Tobacco advertising exposure was interpreting by enquiring if the respondent saw an advertisement about tobacco in a place in the past 30 days.

### 2.5 Data collection and quality control

Before the survey, each investigator participated in a unified training and assessment. During the survey, to ensure the rigor of the survey and the authenticity of the questionnaire, the investigators entered the class and distributed and explained questionnaires in the case of the recusal of teachers. The investigators explained to the students about the anonymity of the survey, the confidentiality of the survey results, and voluntary participation. The investigators also told these students that no answer was *right* or *wrong*. After the investigation, the quality controller performed double entry, cleaned and checked the questionnaire's logic and flow, and excluded unqualified questions.

### 2.6 Statistical analysis

All computations were performed using the SPSS 21.0 complex samples procedure. Multivariate logistic regression was used to explore the factors influencing e-cigarette experimentation and use. As per the testing standards used, α was chosen as 0.05. A weighting factor was applied to each student. The weights corresponding to each student represents the number of students in the total. Final weights = ${\text{W}}_{1}^{*}{\text{W}}_{2}^{*}{\text{W}}_{3}$, where W_1_ represents sampling weights (calculated from the sampling steps in the sampling design); W_2_ represents no-answer weights (no-response adjustment based on responses from monitoring sites, schools, and individuals); and W_3_ represents post-facto stratified correction weights (adjusted for each province by gender and residence population).

## 3 Results

### 3.1 Cigarette experimentation and use

As per the survey results of 2021, 12.11% of students reported having experimented with smoking cigarettes in the past, and the proportion was lower than that in 2019 (13.25%) (*P* < 0.001). From 2019 to 2021, experimented smoking rates showed an overall downward trend. Boys experimented with smoking at a higher rate (16.98%) than girls (6.41%) (*P* < 0.001). Experimented smoking in urban areas was 10.88%, while rural areas was 13.39% (*P* = 0.212). The smoking rates of junior high school students and academic high school students were 9.89% and 11.71%, respectively, while that of vocational high school students was markedly higher at 22.62% (*P* < 0.001). Similar trends were observed in 2019.

In 2021, 3.67% of students were current smokers, which was 4.17% in 2019 (*P* = 0.139), and the smoking rate of boys (5.36%) was higher than girls (1.69%) (*P* < 0.001). In different school types, the smoking rates for students in junior high school, academic high school, and vocational high school were 2.83%, 1.90%, 10.54% (*P* < 0.001). No significant difference in current smoking rates between urban (3.72%) and rural areas (3.61%) was found (*P* = 0.921). Similar results were observed in 2019. The details are presented in Table 2.

**Table 2:** Cigarette experimentation and use among adolescents in Jiangsu.

		**Experimenter**		**Smoker**	
		**2019**	**2021**	**2019**	**2021**
		**% (95% CI)**	**% (95% CI)**	**% (95% CI)**	**% (95% CI)**
Overall		13.25 (10.76–16.21)	12.11 (10.19–14.34)	4.17 (2.96–5.85)	3.67 (2.74–4.89)
Gender	Boys	18.40 (14.83–22.59)	16.98 (14.42–19.89)	6.44 (4.56–9.02)	5.36 (3.93–7.27)
	Girls	7.35 (5.91–9.10)	6.41 (5.08–8.06)	1.59 (1.06–2.37)	1.69 (1.07–2.66)
Residence	Urban	11.86 (9.42–14.82)	10.88 (8.71–13.51)	3.26 (2.36–4.49)	3.72 (2.58–5.34)
	Rural	14.67 (10.69–19.79)	13.39 (10.43–17.03)	5.10 (3.07–8.37)	3.61 (2.28–5.68)
School type	Junior high school	10.94 (7.73–15.28)	9.89 (7.61–12.75)	3.28 (1.86–5.72)	2.83 (1.96–4.07)
	Grade 1	6.45 (4.41–9.35)	6.46 (4.87–8.52)	1.09 (0.49–2.44)	1.23 (0.62–2.42)
	Grade 2	11.51 (7.15–18.00)	10.20 (7.15–14.35)	3.76 (1.38–9.82)	2.56 (1.41–4.59)
	Grade 3	15.53 (10.81–21.81)	13.33 (9.58–18.25)	5.29 (3.17–8.71)	4.88 (3.12–7.54)
	Academic high school	11.49 (8.97–14.61)	11.71 (8.92–15.23)	2.22 (1.54–3.19)	1.90 (1.09–3.29)
	Grade 1	10.80 (7.28–15.73)	12.35 (8.77–17.10)	2.83 (1.51–5.23)	1.34 (0.75–2.39)
	Grade 2	11.14 (7.93–15.42)	9.72 (6.57–14.16)	2.32 (1.46–3.68)	1.74 (0.87–3.43)
	Grade 3	12.64 (9.62–16.43)	13.14 (9.70–17.57)	1.43 (0.78–2.62)	2.77 (1.20–6.27)
	Vocational high school	24.82 (18.87–31.90)	22.16 (17.94–27.03)	10.75 (7.51–15.16)	10.54 (8.05–13.68)
	Grade 1	21.74 (13.83–32.47)	25.48 (17.19–36.03)	8.94 (4.47–17.08)	10.50 (6.86–15.75)
	Grade 2	26.98 (19.79–35.62)	16.89 (10.45–26.14)	14.43 (9.78–20.79)	9.43 (4.83–17.61)
	Grade 3	25.68 (19.67–32.79)	24.05 (18.24–31.00)	8.70 (5.15–14.32)	11.83 (7.59–17.97)

### 3.2 E-cigarette experimentation, use, and awareness

As shown in Table 3, 13.07% of students had experimented with e-cigarettes in 2021, which was higher than 9.34% in 2019 (*P* < 0.001). From 2019 to 2021, experimented e-cigarette rates displayed an overall upward trend. In 2021, the experimentation rate among boys (17.77%) was higher than girls (7.56%) (*P* < 0.001). Junior high school was 11.10% and academic high school was 11.60%, vocational high school (24.07%) was higher than both (*P* < 0.001). Similar results were also seen in current e-cigarette using rates. No significant difference in current using rates between urban (12.22%) and rural areas (13.95%) was found (*P* = 0.411). Whether cigarette or e-cigarettes, the most obvious change in rate was among junior school students, with a clear upward trend from grade 1 to grade 3. In 2021, cigarette experimenters from grade 1 to grade 3: 6.46%, 10.20%, 13.33%; e-cigarette experimenters from grade 1 to grade 3: 7.59%, 9.98%, 16.18%. In contrast, no clear trend of change was found with age when it came to academic high school and vocational students.

**Table 3:** E-cigarette experimentation and use among adolescents in Jiangsu.

		**Experimenter**		**Smoker**		**Awareness**	
		**2019**	**2021**	**2019**	**2021**	**2019**	**2021**
		**% (95% CI)**	**% (95% CI)**	**% (95% CI)**	**% (95% CI)**	**% (95% CI)**	**% (95% CI)**
Overall		9.34 (7.87–11.04)	13.07 (11.07–15.36)	2.23 (1.65–3.00)	3.74 (2.48–4.58)	76.72 (73.97–79.26)	89.38 (87.64–90.91)
Gender	Boys	14.30 (11.96–17.01)	17.77 (15.08–20.82)	3.43 (2.48–4.73)	5.15 (4.00–6.60)	79.77 (77.34–82.00)	89.32 (87.45–90.94)
	Girls	3.64 (2.93–4.52)	7.56 (6.15–9.25)	0.84 (0.54–1.31)	2.10 (1.47–2.99)	73.22 (69.33–76.78)	89.46 (87.36–91.24)
Residence	Urban	10.11 (8.27–12.31)	12.22 (10.10–14.70)	2.01 (1.44–2.80)	3.38 (2.48–4.58)	79.48 (74.91–83.41)	91.17 (89.38–92.68)
	Rural	8.55 (6.35–11.43)	13.95 (10.69–18.02)	2.45 (1.54–3.88)	4.13 (2.90–5.84)	73.91 (70.36–77.18)	87.52 (84.67–89.91)
School type	Junior high school	8.38 (6.63–10.53)	11.10 (8.71–14.04)	2.40 (1.60–3.58)	3.26 (2.34–4.53)	69.95 (66.37–73.30)	85.29 (82.74–87.51)
	Grade 1	4.40 (2.94–6.56)	7.59 (6.00–9.55)	1.65 (0.93–2.89)	1.75 (1.09–2.79)	56.56 (51.00–61.95)	77.61 (74.15–80.72)
	Grade 2	8.97 (6.10–13.01)	9.98 (7.23–13.61)	2.60 (1.30–5.12)	2.95 (1.88–4.62)	72.57 (68.21–76.53)	87.34 (83.90–90.13)
	Grade 3	12.31 (10.09–14.95)	16.18 (12.27–21.04)	3.06 (2.23–4.18)	5.26 (3.39–8.09)	82.52 (79.28–85.36)	91.52 (88.06–94.05)
	Academic high school	7.00 (5.67–8.62)	11.60 (9.20–14.54)	0.78 (0.54–1.14)	2.45 (1.47–4.08)	85.36 (82.33–87.94)	95.31 (93.94–96.38)
	Grade 1	7.08 (5.03–9.88)	12.19 (9.47–15.56)	0.84 (0.45–1.57)	1.65 (1.02–2.65)	83.36 (79.86–86.35)	94.63 (92.46–96.20)
	Grade 2	6.70 (5.33–8.39)	9.58 (7.00–12.97)	0.63 (0.32–1.27)	2.32 (1.19–4.48)	85.42 (82.33–88.04)	95.87 (93.57–97.38)
	Grade 3	7.22 (5.24–9.86)	12.19 (9.47–15.56)	0.88 (0.51–1.50)	3.60 (1.85–6.88)	87.56 (81.67–91.75)	95.52 (94.02–96.65)
	Vocational high school	16.79 (12.69–21.89)	24.07 (20.37–28.20)	3.94 (2.43–6.32)	8.18 (6.71–9.94)	88.10 (85.56–90.24)	95.33 (93.94–96.41)
	Grade 1	17.57 (11.16–26.57)	27.39 (20.81–35.13)	4.83 (1.84–12.07)	9.95 (6.07–15.89)	87.27 (82.08–91.13)	96.07 (94.15–97.38)
	Grade 2	18.91 (13.16–26.40)	20.41 (14.22–28.39)	3.60 (2.00–6.39)	6.78 (4.36–10.39)	88.59 (78.86–94.17)	94.37 (92.18–95.98)
	Grade 3	13.77 (8.81–20.89)	24.14 (19.13–29.98)	3.38 (1.52–7.35)	7.62 (5.74–10.06)	88.42 (78.81–94.01)	95.51 (92.72–97.25)

The rate of e-cigarette use was 3.74% in 2021, higher than 2.23% in 2019 (*P* < 0.001). From 2019 to 2021, e-cigarette experimentation displayed an overall upward trend in various populations. In 2021, the rate of e-cigarette experimentation among boys was 5.15%, which was significantly higher than 2.10% among girls (*P* < 0.001). The rates among urban and rural populations were 3.38% and 4.13% (*P* = 0.390), respectively, and among junior high school, academic high school, and vocational high school students were 3.26, 2.45, and 8.18% (*P* < 0.001), respectively.

We also surveyed students about their awareness of e-cigarettes. Among the surveyed students, 89.38% reported they had heard of e-cigarettes in 2021, while it was only 76.72% in 2019 (*P* < 0.001). No significant differences were found between the sexes and residences. Academic high school (95.31%) and vocational high school (95.53%) students were more aware about the same than junior high school (85.29%) (*P* < 0.001).

**Figure 1:**
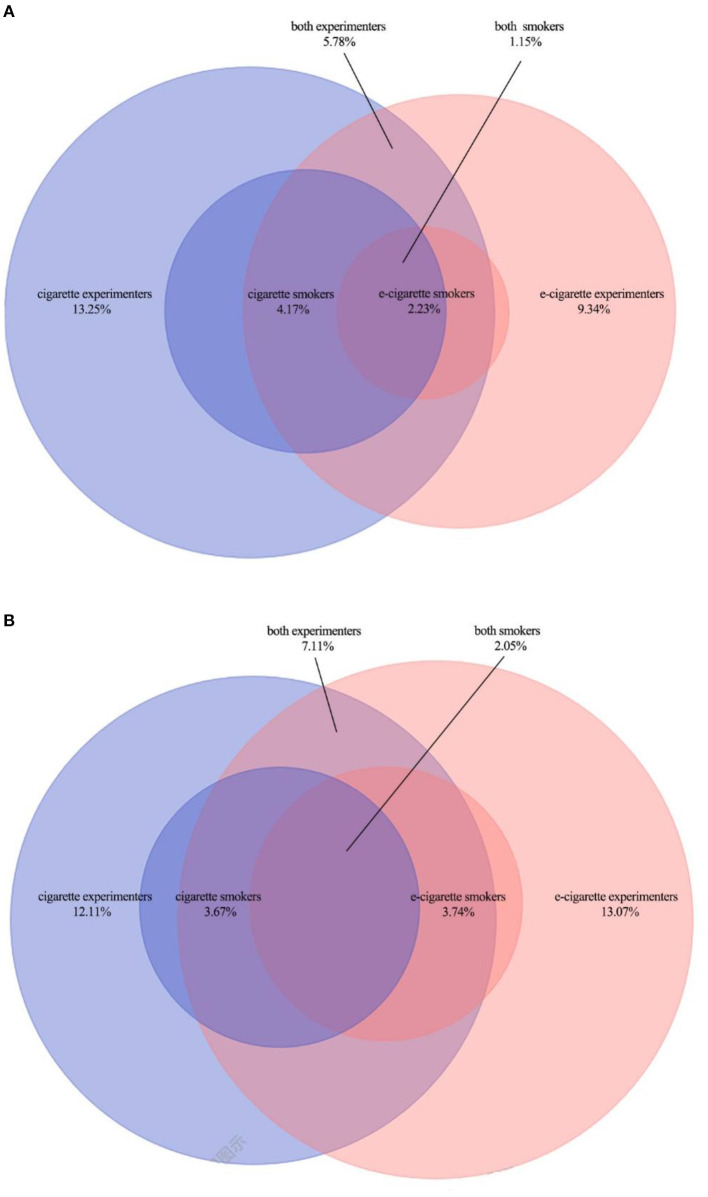
**(A)** Tobacco experimentation and use among adolescents in 2019. **(B)** Tobacco experimentation and use among adolescents in 2021. Sections in blue indicate cigarette smokers, includes light colored large round “cigarette experimenters” and dark colored small round “cigarette experimenters”. Sections in orange indicate e-cigarette smokers, includes light colored large round “e-cigarette experimenters” and dark colored small round “e-cigarette experimenters”. The blue and orange cross section “both smokers” indicates who smoke both cigarettes and e-cigarettes, “both experimenters” indicates who experiment both cigarettes and e-cigarettes.

Of all students surveyed in 2019, the ratio of simultaneous experiments smoking cigarettes and e-cigarettes was 5.78%, which rose to 7.11% in 2021 (*P* < 0.001). The ratio of simultaneous current smoking cigarettes and e-cigarettes was 1.15% in 2019, which rose to 2.05% in 2021 (*P* < 0.001) (Figure 1).

### 3.3 Single-factor analysis of e-cigarette experimentation and use

We explored the factors influencing e-cigarette experimentation and use among adolescents from individual characteristics, along with social psychological, and environmental–structural factors. The percentage of experimented smokers in 2021 is higher than in 2019 for all indicators. The current e-cigarette use rate also showed the same pattern.

In individual characteristics, in addition to the previously mentioned gender, residence, and age, the rates of e-cigarette experimentation increased significantly from 8.52% to 24.03% as the pocket money increased (*P* < 0.001). Of these, the effect of cigarette experimentation on students' e-cigarette using is the largest among the factors and shows a significant upward trend from 2019 to 2021 [experimentation rate in 2021 (58.82%) > rate in 2019 (43.74%) (*P* < 0.001); e-cigarette using rate in 2021 (22.62%) > rate in 2019 (11.52%)] (*P* < 0.001).

In social psychological factors, higher e-cigarette experimentation and use rates among students who are exposed to SHS. Exposure environments included home, indoors, outdoors, public transport, and the school. Students exposed to tobacco advertising had higher e-cigarette experimentation and use rates. Tobacco advertising exposure included smoking footage on TV, tobacco advertisements on the Internet, being offered free tobacco products. Students who had received antitobacco media messages and learned about tobacco in class, considered SHS harmful. They found it hard to quit using e-cigarette and had lower e-cigarette experimentation and use rates. All of the above *P* < 0.05.

In environmental–structural factors, when surrounded by people (parents, friends, or teachers) who smoke, students were more likely to experiment and use an e-cigarette. Those who found smoking more attractive and comfortable would use e-cigarettes provided by friends and want to use e-cigarettes in the future. They had a higher propensity to use e-cigarettes, and thus, their e-cigarette experimentation and use rates were higher.

The details are presented in Table 4.

**Table 4:** Association among individual characteristics, social psychological, and environmental–structural factors in terms of prevalence of e-cigarette experimentation and use among adolescents in Jiangsu.

		**Experimenter**		**Smoker**	
		**2019**	**2021**	**2019**	**2021**
		**% (95% CI)**	**% (95% CI)**	**% (95% CI)**	**% (95% CI)**
Gender	Boys	14.30 (11.96–17.01)	17.77 (15.08–20.82)	3.43 (2.48–4.73)	5.15 (4.00–6.60)
	Girls	3.64 (2.93–4.52)	7.56 (6.15–9.25)	0.84 (0.54–1.31)	2.10 (1.47–2.99)
Residence	Urban	10.11 (8.27–12.31)	12.22 (10.10–14.70)	2.01 (1.44–2.80)	3.38 (2.48–4.58)
	Rural	8.55 (6.35–11.43)	13.95 (10.69–18.02)	2.45 (1.54–3.88)	4.13 (2.90–5.84)
School type	Junior high school	8.38 (6.63–10.53)	11.10 (8.71–14.04)	2.40 (1.60–3.58)	3.26 (2.34–4.53)
	Academic high school	7.00 (5.67–8.62)	11.60 (9.20–14.54)	0.78 (0.54–1.14)	2.45 (1.47–4.08)
	Vocational high school	16.79 (12.69–21.89)	24.07 (20.37–28.20)	3.94 (2.43–6.32)	8.18 (6.71–9.94)
Pocket money (RMB)	≤ 20	6.14 (4.93–7.63)	8.52 (6.92–10.45)	1.50 (0.96–2.33)	2.01 (1.42–2.83)
	>20, ≤ 50	11.27 (9.29–13.60)	16.08 (13.57–18.96)	2.73 (1.79–4.13)	4.55 (3.59–5.76)
	>50	14.47 (11.88–17.50)	24.03 (20.49–27.97)	3.32 (2.38–4.63)	8.67 (6.37–11.69)
Cigarette experimentation	Yes	43.74 (39.83–47.72)	58.82 (53.12–64.30)	11.52 (8.82–14.89)	22.62 (18.80–26.97)
	No	4.08 (3.51–4.75)	6.77 (5.92–7.74)	0.82 (0.60–1.14)	1.14 (0.94–1.39)
Exposure to SHS at home	Yes	14.38 (12.04–17.09)	21.57 (18.02–25.59)	4.38 (3.15–6.06)	8.36 (6.39–10.86)
	No	6.79 (5.61–8.20)	10.02 (8.52–11.75)	1.15 (0.80–1.65)	2.09 (1.64–2.65)
Exposure to SHS indoors	Yes	14.59 (12.30–17.23)	21.94 (18.17–26.25)	3.65 (2.65–5.01)	7.43 (5.57–9.84)
	No	4.50 (3.72–5.42)	8.01 (6.84–9.35)	0.92 (0.63–1.35)	1.64 (1.29–2.08)
Exposure to SHS outdoors	Yes	14.04 (11.79–16.65)	20.52 (17.45–23.96)	3.50 (2.53–4.83)	7.09 (5.52–9.05)
	No	4.45 (3.67–5.40)	8.04 (6.69–9.63)	0.91 (0.63–1.30)	1.48 (1.14–1.93)
Exposure to SHS on public transport^*^	Yes	21.65 (17.89–25.96)	33.10 (28.06–38.57)	7.58 (5.07–11.17)	14.68 (10.97–19.37)
	No	8.23 (6.40–10.53)	14.36 (11.62–17.62)	1.95 (1.37–2.77)	4.42 (3.32–5.87)
Exposure to SHS in school	Yes	15.46 (13.20–18.03)	21.81 (18.73–25.23)	3.78 (2.89–4.91)	6.63 (5.11–8.57)
	No	4.39 (3.59–5.34)	7.46 (6.13–9.06)	0.98 (0.62–1.56)	1.89 (1.44–2.47)
Exposure to smoking footage on TV	Yes	12.89 (11.04–15.01)	18.60 (15.69–21.92)	3.28 (2.42–4.42)	5.88 (4.61–7.49)
	No	5.35 (4.35–6.56)	8.00 (6.80–9.40)	1.05 (0.67–1.62)	1.78 (1.37–2.32)
Exposure to tobacco ads on the Internet	Yes	13.25 (11.23–15.58)	21.12 (18.35–24.18)	3.45 (2.58–4.61)	8.40 (6.77–10.37)
	No	8.64 (7.15–10.39)	11.70 (9.75–13.98)	2.00 (1.38–2.91)	2.95 (2.23–3.89)
Be offered free tobacco products	Yes	19.49 (13.32–27.62)	29.77 (24.09–36.15)	6.09 (2.78–12.78)	12.99 (8.74–18.89)
	No	9.21 (7.74–10.93)	12.77 (10.78–15.06)	2.18 (1.60–2.96)	3.58 (2.82–4.54)
Exposure to antitobacco media messages in past 30d	Yes	8.53 (6.98–10.38)	12.30 (10.42–14.47)	1.90 (1.32–2.72)	3.65 (2.92–4.57)
	No	10.61 (8.92–12.57)	14.37 (11.97–17.16)	2.74 (2.03–3.69)	3.91 (2.88–5.28)
Exposed to e-cigarette advertising	Yes	14.91 (12.39–17.84)	14.27 (11.79–17.17)	5.14 (3.78–6.96)	5.40 (4.24–6.85)
	No	7.54 (6.37–8.91)	12.22 (10.45–14.25)	1.28 (0.94–1.76)	2.58 (1.92–3.47)
Knowledge about the harm of SHS	Yes	8.81 (7.38–10.49)	11.82 (10.01–13.91)	1.78 (1.37–2.32)	2.92 (2.30–3.69)
	No	11.16 (9.11–13.60)	16.26 (13.55–19.40)	3.76 (2.54–5.54)	5.86 (4.47–7.63)
Learned about tobacco in class	Yes	6.96 (5.55–8.70)	11.28 (9.21–13.74)	1.76 (1.26–2.45)	3.69 (2.80–4.85)
	No	10.93 (9.30–12.82)	14.16 (12.03–16.61)	2.54 (1.85–3.48)	3.77 (2.93–4.85)
Believe it is hard to quit smoking	Yes	7.61 (6.39–9.04)	10.92 (9.27–12.81)	1.98 (1.48–2.65)	3.00 (2.35–3.81)
	No	16.25 (13.77–19.06)	23.29 (19.43–27.66)	3.21 (2.24–4.59)	7.30 (5.56–9.53)
Parents smoking	Yes (father or mother or both)	11.55 (9.98–13.34)	16.44 (13.95–19.27)	2.92 (2.20–3.86)	5.37 (4.20–6.85)
	No	7.17 (5.68–9.02)	9.69 (8.06–11.59)	1.55 (0.99–2.42)	2.11 (1.56–2.84)
Closest friends smoking	Yes	24.28 (21.74–27.02)	36.31 (32.87–39.89)	6.37 (4.89–8.26)	12.98 (10.94–15.34)
	No	3.44 (2.86–4.14)	4.95 (4.19–5.84)	0.60 (0.41–0.85)	0.52 (0.36–0.74)
Teachers smoking	Yes	15.13 (13.11–17.41)	20.80 (17.79–24.16)	3.83 (2.91–5.03)	6.22 (4.81–8.00)
	No	4.60 (3.70–5.71)	7.39 (6.02–9.06)	0.90 (0.61–1.33)	1.93 (1.41–2.62)
Believe smoking makes youth look more or less attractive	More	28.70 (22.93–35.25)	26.71 (21.00–33.30)	9.07 (6.76–12.08)	11.64 (8.69–15.42)
	No difference	22.02 (18.49–26.02)	20.56 (17.17–24.43)	3.69 (2.61–5.18)	6.94 (5.40–8.88)
	Less	8.97 (7.17–11.17)	9.27 (7.90–10.85)	1.11 (0.79–1.55)	1.93 (1.43–2.60)
Believe tobacco helps youth feel more comfortable in social situations	More comfortable	35.16 (29.80–40.91)	35.51 (29.43–42.10)	14.17 (10.82–18.35)	18.10 (14.15–22.87)
	No difference	27.84 (23.50–32.64)	32.56 (27.32–38.27)	8.22 (6.15–10.90)	12.90 (10.27–16.08)
	More uncomfortable	6.57 (5.54–7.77)	9.39 (7.97–11.03)	1.18 (0.82–1.69)	1.79 (1.35–2.37)
Will use an e-cigarette product if your best friend offered	Yes	57.10 (52.74–61.35)	68.60 (62.65–74.01)	17.00 (13.39–21.35)	29.59 (25.26–34.31)
	No	4.00 (3.40–4.71)	6.66 (5.76–7.69)	0.61 (0.42–0.89)	0.83 (0.64–1.08)
Next 12 months, do you think you will use any e-cigarette	Yes	63.65 (58.61–68.40)	73.59 (68.59–78.06)	27.51 (21.85–34.00)	41.85 (37.30–46.54)
	No	6.15 (5.26–7.19)	8.64 (7.41–10.06)	0.90 (0.65–1.24)	1.13 (0.84–1.53)

Exposure to SHS on public transport^*^: Include in the survey only those who have taken transportation in the past 7 days.

### 3.4 Determinants of e-cigarette experimentation using a multivariate logistic regression model

**Figure 2:**
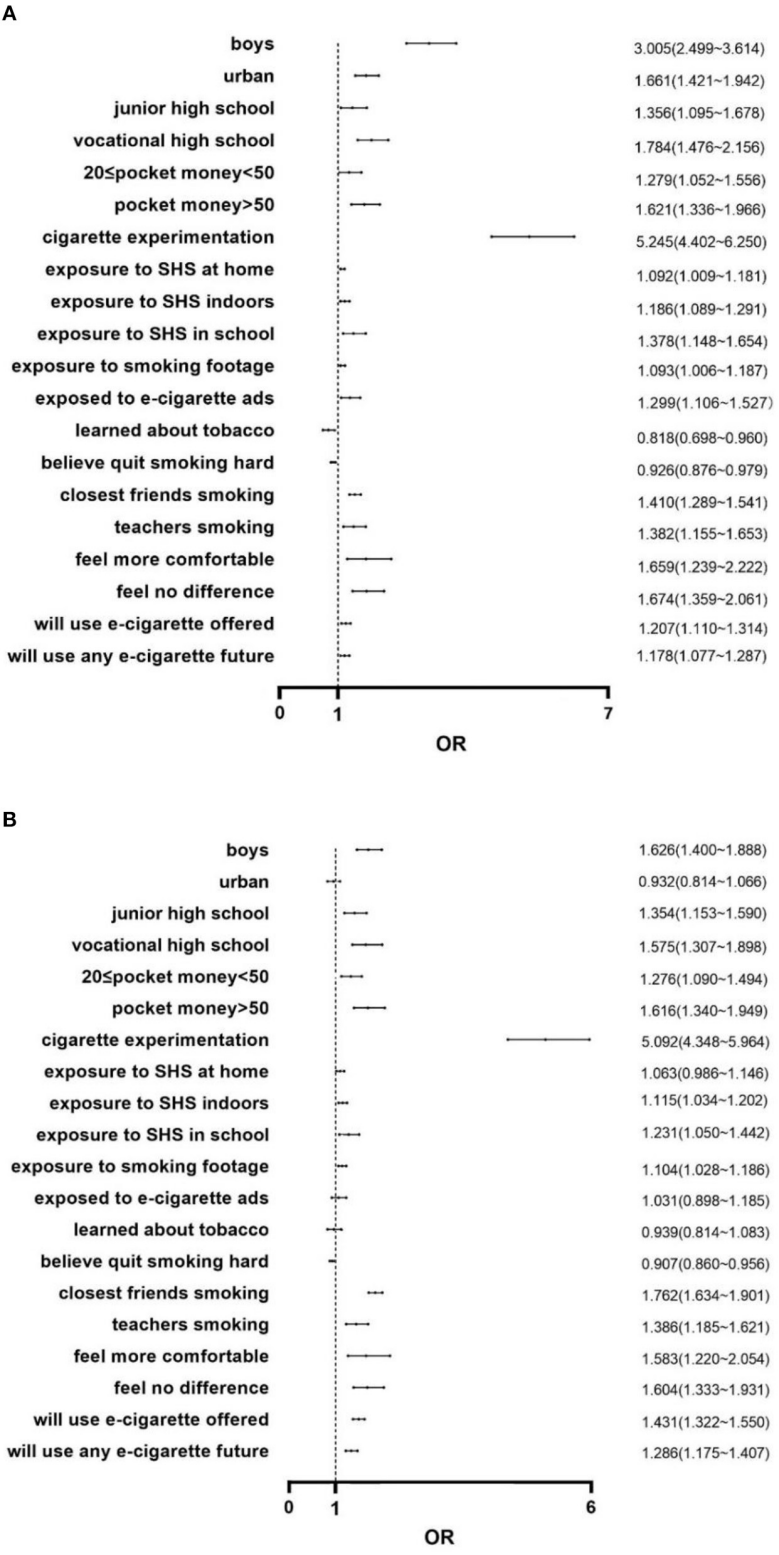
**(A)** Determinants of e-cigarette experimentation among adolescents in Jiangsu China in 2019. **(B)** Determinants of e-cigarette experimentation among adolescents in Jiangsu China in 2021.

We considered e-cigarette experimentation the dependent variable to build a multivariate logistic regression model for 2019 and 2021. As evident in Figure 2, certain variables remained statistically significant in determining the e-cigarette experimentation and exhibited similar patterns in both 2019 and 2021. For 2021, the variable most strongly associated with e-cigarette experimentation was cigarette experimentation (OR = 5.092). Boys were more likely to experiment with e-cigarettes than girls (OR = 1.626). Compared with academic high school students, junior high school (OR = 1.354) and vocational high school (OR = 1.575) students preferred experimenting with e-cigarettes. Participants were more likely to experiment with e-cigarettes if they had more pocket money per week (OR_1_ = 1.276, OR_2_ = 1.616). Those who were exposed to an SHS environment (home, OR = 1.063; indoors, OR = 1.115; school, OR=1.231) were more likely to experiment with e-cigarettes. Students exposed to smoking advertisements had higher rates of experimenting with e-cigarettes (OR = 1.104). Students were less likely to experiment with e-cigarettes if they believed it was hard to quit (OR = 0.907). These students were more likely to experiment when friends (OR = 1.762) or teachers (OR = 1.386) around them smoked. Students who believed that smoking helped them feel more comfortable or no difference rather than more uncomfortable in social situations were more likely to experiment (OR_1_ = 1.583, OR_2_ = 1.604). Respondents were also more likely to use e-cigarettes if offered by a close friend (OR = 1.431) or if they intended to use an e-cigarette product in the next 12 months (OR = 1.286). In 2021, we did not find a difference in residence, exposure to e-cigarette advertising, and learning about tobacco in class. In 2019, urban students had higher e-cigarette experimentation rates than their rural counterparts (OR = 1.661). Students were more likely to experiment if they were exposed to e-cigarette advertising (OR = 1.299). Learning about tobacco in class was a protective factor (OR = 0.818).

### 3.5 Determinants of e-cigarette use by a multivariate logistic regression model

**Figure 3:**
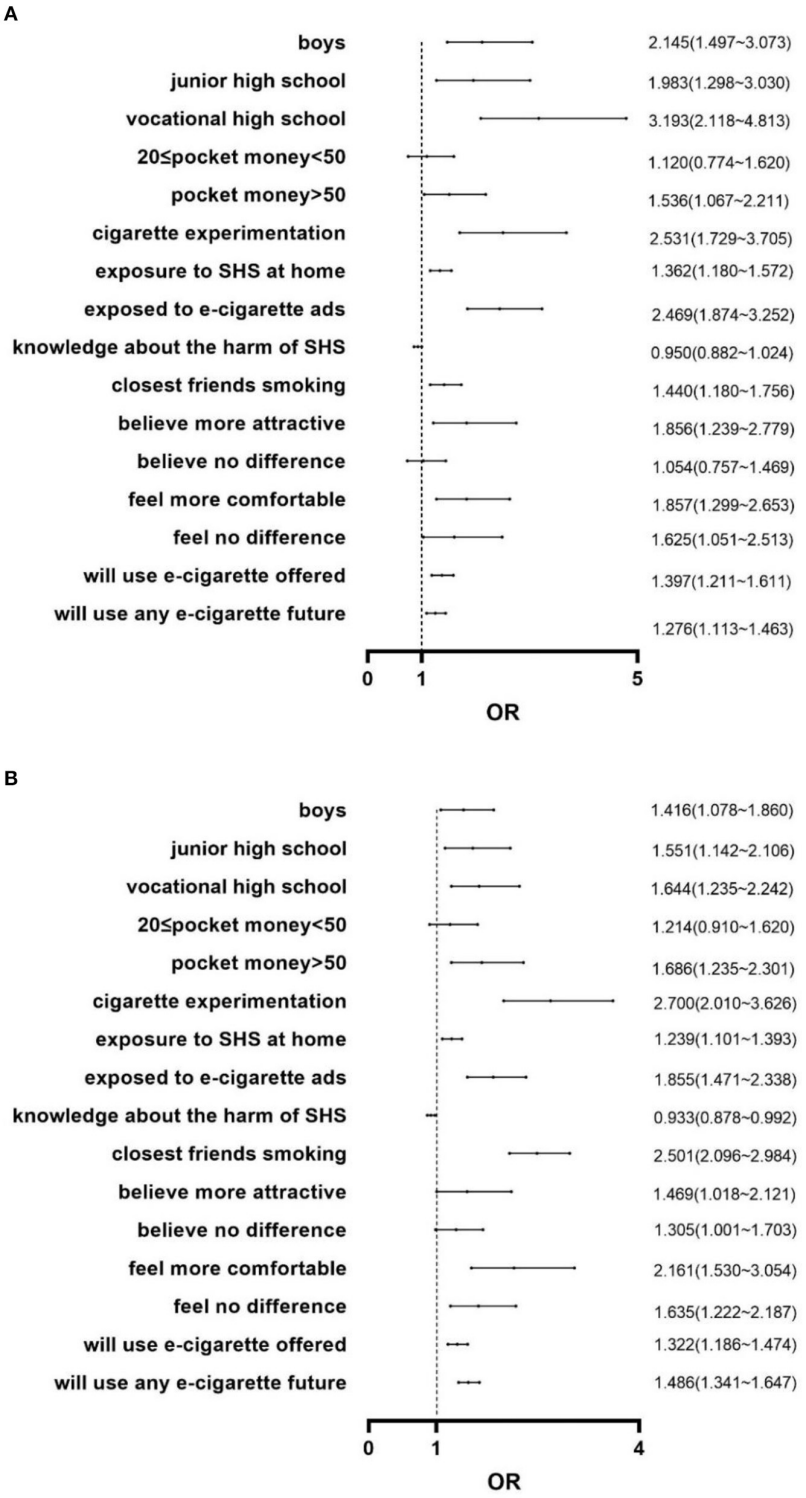
**(A)** Determinants of e-cigarette use among adolescents in Jiangsu in 2019. **(B)** Determinants of e-cigarette use among adolescents in Jiangsu in 2021.

We built a multivariate logistic regression model for 2019 and 2021 using the same method as above, making e-cigarette use the dependent variable. As shown in Figure 3, both years reported similar results. For 2021, the variable most strongly associated with e-cigarette experimentation was once again cigarette experimentation (OR = 2.700). Boys were more likely to use e-cigarettes than girls (OR = 1.416). Compared with academic high school students, junior high school (OR = 1.551) and vocational high school (OR = 1.644) students preferred using an e-cigarette. Respondents were more likely to use an e-cigarette if they had more pocket money per week (OR_1_ = 1.214, OR_2_ = 1.686). Those who were exposed to SHS at home (OR = 1.239) were more likely to use an e-cigarette. Students were more likely to use an e-cigarette if they were exposed to e-cigarette advertising (OR = 1.855). Students who considered SHS harmful were less likely to use an e-cigarette (OR = 0.933). Students were more likely to experiment when their friends (OR = 2.501) around them smoked. Students who felt that smoking made them look more attractive or no difference rather than less attractive (OR_1_ = 1.469, OR_2_ = 1.305) or believed that smoking helped them feel more comfortable or no difference rather than more uncomfortable in social situations (OR_1_ = 2.161, OR_2_ = 1.635) were more likely to use an e-cigarette. Respondents were also more likely to use e-cigarettes if offered by a close friend (OR = 1.322) or if they intend to use an e-cigarette product in the next 12 months (OR = 1.486).

## 4 Discussion

Cigarette experimentation and use rates in Jiangsu Province in 2021 were respectively 12.11% and 3.67%, which were lower by 1.14% and 0.50% from 2019. The two rates were lower than China in 2019 (junior high school, 12.9% and 3.9%; senior high school, 24.5% and 8.6%), lower than Fujian Province with 14.5% and 4.4%, Henan Province with 23.8% and 7.5%, Liaoning Province with 13.6% and 4.8%, and lower than Americans with 24.1% and 9.3% (2). These data indicate that the smoking rate among adolescents in Jiangsu Province was lower than China's national average and developed countries. The smoking rate in Jiangsu Province is relatively low. However, it does not imply that tobacco use among adolescents has improved. E-cigarette experimentation and use rate in Jiangsu Province in 2021 were 13.07% and 3.74%, respectively, both higher than cigarettes, and the rates increased by 3.73% and 1.51% from 2019. The rise in e-cigarettes is greater than the decline in cigarettes. Jiangsu's e-cigarette use rate was below the national average in 2019 (junior high school, 2.7%; senior high school, 3.0%). These statistics suggest that the state of youth tobacco use today is not optimistic. While cigarette use has declined, more youth are aware of e-cigarettes and have started to use them. The e-cigarette awareness rate in Jiangsu rose rapidly from 76.72% in 2019 to 89.38% in 2021. It was only 45.0% in 2013 in China, and e-cigarette use among adolescents was only 1.2% in 2013 in China (16). Therefore, it is likely that e-cigarettes will replace cigarettes and occupy the Chinese tobacco market in the future (20).

From the above data, cigarette experimentation and use rates fell by 1.14% and 0.50%. It could be ascribed to the good economic development of Jiangsu. The economic situation is stable, and thus, more human and material resources can be invested in the construction of tobacco control. Jiangsu has rich medical and health resources and health education resources. Many smoke-free government agencies, smoke-free schools, and smoke-free community construction have been established, which has formed a long-term mechanism. Various tobacco control efforts have also yielded fruitful outcomes. However, Jiangsu also has significant shortcomings compared with cities such as Beijing and Shanghai. Owing to no tobacco control legislation in Jiangsu and no restrictions on the use of e-cigarettes in public places, one can smoke anywhere outdoors.

While cigarettes are an old type of tobacco product, e-cigarettes are a new type. In the past, many adolescents had to use e-cigarettes owing to inadequate access of cigarettes. Both cigarettes and e-cigarettes contain addictive nicotine, which contributes to the fact that many e-cigarette smokers evolved from cigarette smokers (21). E-cigarette use is an independent risk factor for smoking initiation (16, 22). In other words, more non-smoking adolescents first time tried using with an e-cigarette, which is the same as the results of this study.

Men have always been the focus of tobacco control efforts for the high smoking rate. However, the female population also has a large number of smokers, who were vulnerable to exposure to the SHS environment (23). E-cigarette experimentation and use among girls in this study increased from 3.64% and 0.84% in 2019 to 7.56% and 2.10% in 2021. The increase is much greater than that of male students. Therefore, adolescent tobacco control efforts should also focus on girls in the future. In our study, both cigarette and e-cigarette use were higher in rural areas than in urban, which is similar to that of the United States (24). Rural smokers have higher daily cigarette consumption (23). As e-cigarettes become popular, access to them in rural areas has become more convenient. Now, e-cigarettes have become the most common tobacco product among students with higher rates of use in rural areas (25, 26). The study found that students with more pocket money have higher e-cigarette using rates. The more the pocket money, the easier it is to buy e-cigarettes, and Chinese stores do not turn away minors who come to buy cigarettes (27).

E-cigarette using rates increase with age (28). Our study was conducted by grade level rather than age as the variable. In China, students in the same grade are exposed to more similar things, and thus, such a study would be more meaningful. Vocational high school students' e-cigarette use rate was 8.18% in 2021, significantly higher than junior school (3.26%) and academic high school students (2.45%). The same trend was observed in the rate of e-cigarette experimentation. Smoking behavior is correlated with academic performance. Smoking rates are higher among students with lower academic performance (29), who are more likely to receive e-cigarette messages (28, 30). In China, every junior high school student is required to take academic tests. Students with better academic performance will enter academic high school, while those with lower academic performance enter vocational high school. High smoking rates among vocational high school students may also be linked to poor school smoke-free practices. From the data, we can find that the smoking rate is rising fastest from grade 1 to grade 3 in junior high school. Students are sensitive to tobacco exposure in this age group. They have not established a correct perception of tobacco hazards and are influenced by curiosity and imitation to experiment with smoking (31).

SHS is a mixture of smoke from the burning end of tobacco and smoke breathed out from smoker. It can also cause respiratory disease, cardiovascular disease, and cancer (32). SHS from e-cigarettes is more sweet-smelling (33), which may stimulate the curiosity of teenagers, causing them not to dodge. Adolescents themselves lack the ability to avoid SHS, contributing to their tolerance of smoking behavior and their own exposure to SHS. This survey shows that adolescents are commonly exposed to SHS. Those who are exposed to SHS, especially at home, are more likely to smoke. High rates of SHS exposure are even among adolescents who never tried smoking (34). Since adolescents are in a state of physical and psychological development, they are vulnerable to the effects of SHS. It is imperative to protect adolescents from the perils of SHS during critical developmental stages and prevent the burden of disease that lasts into adulthood (35). Studies have shown that smoking ban (36), community anti-smoking interventions, and smoke-free home rules can substantially reduce SHS exposure (37). However, taking action on tobacco control requires an enforcement authority and strong enough enforcement. Most areas in China, including Jiangsu, do not yet have regulations to control smoking in public places. Therefore, it is necessary to strengthen tobacco control regulation and legislation and promote the construction of smoke-free places.

This study found that students exposed to tobacco advertising, tobacco retailing, and smoking footage are more likely to use e-cigarettes. Exposure to tobacco advertising and promotion may stimulate adolescents' curiosity about smoking and alter their attitudes toward smoking, contributing to the acceptance of smoking (38, 39). Nowadays one can see a large number of e-cigarette retail and experience stores in shopping malls compared with the previous years. The Advertising Law of the People's Republic of China was revised in 2015, prohibiting tobacco advertising to youth in mass media and public places (34). We found that smoking scenes are rarely seen on TV and online videos today thanks to the restrictions imposed by the Broadcasting Authority. However, tobacco advertising is still visible in tobacco retail stores, and no ban on the sale of cigarettes to minors has been imposed.

Adolescent smoking behavior is strongly influenced by environmental–structural factors, which include the smoking situation of people around the adolescent individual, including parents, friends, and teachers, among others. We found no effect of parental smoking on adolescents through our multifactorial analysis. Adolescents do not show their curiosity about smoking to their parents for they are in adolescence with a rebellious mentality. The vast majority of adolescents who smoke will avoid their parents. Rather than parents, closest friends smoking has a strong impact on adolescents, (e-cigarette experimentation, OR = 1.966; e-cigarette use, OR = 2.779). Adolescents at the developmental stage are susceptible to peer influence on their thoughts and behavior. Adolescents whose best friends smoke may have higher rates of experimenting with smoking (4, 5). The peer effects can promote smoking behavior; smokers are attracted to each other, which then reinforces adolescents' willingness to continue smoking (40). E-cigarette use can activate behavior and cognition with increased positive expectations of smoking and draw closer to peers (41). Therefore, we should help young people develop the right attitude toward tobacco. School tobacco control efforts need to focus on student smoking groups. Inadequate environmental–structural support for tobacco control is a major risk factor for tobacco initiation among adolescents. Tobacco control strategies should focus not only on the community but also on the school and home.

Therefore, health education on the dangers of tobacco should be widely carried out in schools to popularize knowledge about tobacco, prohibit staff and students from smoking on school grounds, incorporate peer education into tobacco control publicity and education for young people, and work together with parents and the community to build smoke-free campuses, smoke-free homes and smoke-free communities, and so on. We should improve e-cigarette management methods and policies and regulations, incorporate e-cigarette tobacco control into the management of the Tobacco Monopoly Law, strengthen the publicity and education on the health hazards of e-cigarettes, restrict access to e-cigarettes for young people, and reduce e-cigarette brand advertisements and flavor promotions.

Our survey also has several limitations. First, we obtained data via a questionnaire without testing biomarkers. Second, we did not explore the relationship between e-cigarette use and tobacco access in adolescents. It can be achieved through a survey of tobacco retail outlets around schools in the future. Third, we did not investigate students' academic performance. We can add questions about academic performance to the questionnaire in future surveys. Fourth, we did not give adolescents preferred e-cigarette brands and flavors. Fifth, we could not explore the factors influencing the amount and frequency of smoking among adolescents. Finally, our findings only represent the situation in Jiangsu and do not reflect the overall tobacco use in China, such as the western region, where the smoking rate is markedly higher.

The health hazards of tobacco are a major public health problem. China is the world's largest consumer of tobacco with a huge annual burden of disease. Preventing health hazards caused by smoking can not only improve the health of the entire population but also reduce the consumption of medical resources. It is also of great benefit to economic interests. Therefore, tobacco control action is imminent. We should closely monitor changes in adolescent e-cigarette use. We need to focus tobacco control efforts on health education, popularize tobacco knowledge, and improve youth attitudes toward tobacco. It is necessary to introduce anti-smoking legislation in public places, reduce adolescent secondhand smoke exposure, and strengthen control of tobacco advertising, in addition to creating a smoke-free environment that is suitable for healthy adolescent growth.

## Data availability statement

The original contributions presented in the study are included in the article/Supplementary material, further inquiries can be directed to the corresponding author.

## Ethics statement

Ethical approval was not required for the study involving human samples in accordance with the local legislation and institutional requirements. Written informed consent for participation in this study was provided by the participants' legal guardians/next of kin.

## Author contributions

JF wrote the article. TM: Writing—original draft, data curation, formal analysis, and project administration. SZ: writing—review & editing, supervision, and validation. YX: writing—review & editing, methodology, and funding acquisition. CQ designed the survey. All authors contributed to the article and approved the submitted version.
