# Supplementary material for: Comparative analysis of e-cigarette prevalence and influencing factors among adolescents in Jiangsu Province, China
Source: Front Public Health. 2023 Dec 1;11:1221334. doi: 10.3389/fpubh.2023.1221334 (PMC10722425; doi:10.3389/fpubh.2023.1221334)
Supplement: Supplementary file 1 [file Data_Sheet_1.PDF]

2019 年中国青少年烟草流行调查问卷

□□□监测点编码 □□学校编码 □学校类型 □年级 □□个人编码

_______________省（自治区、直辖市）_____________县（市、区）

学校名称 ________________________________

学校类型 ________________（初中/高中/职高）

所在年级：___年级　　　　所在班级：____ 班

调查员签名____________　　　　调查日期____________

质控员签名____________　　　　质控日期____________

你好！国家卫生健康委正在开展全国青少年吸烟相关行为调查，你被选中参加本次调查。调查采用不记名方式，你的回答结果将严格保密，包括对你的同学、老师和家长。你的参与对我们了解真实的情况非常重要，问题的答案没有对错之分，我们只使用汇总信息，不会使用你个人的信息。调查结果将被作为制定卫生健康相关政策的重要依据。你若同意参加本次调查，请自己填写问卷内容；若不同意参加，可将空白问卷交回。感谢你的配合和支持！

（　）1.你多大了，请回答周岁？

① 11 岁及以下

② 12 岁

③ 13 岁

④ 14 岁

⑤ 15 岁

⑥ 16 岁

⑦ 17 岁

⑧ 18 岁

⑨ 19 岁

⑩ 20 岁及以上

（ ）2. 你的性别 ①男 ② 女

（ ）3. 你所在的年级

① 一年级 ②二年级 ③三年级

（ ）4. 平均在一周内，你有多少可以自己支配的钱（不管你怎么花）？

① 我通常没有钱

② 少于或等于 10 元

③ 11～20 元

④ 21～30 元

⑤ 31～40 元

⑥ 41～50 元

⑦ 超过 50 元

（ ）5. 你是否尝试过吸卷烟，即使是一、两口？

① 是 ② 否

（ ）6. 你第一次尝试吸卷烟时的年龄有多大（请回答周岁）？

① 我从未尝试过吸烟

② 7 岁或以下

③ 8 岁或 9 岁

④ 10 岁或 11 岁

⑤ 12 岁或 13 岁

⑥　14 岁或 15 岁

⑦　16 岁或以上

（　）7. 在**过去 30 天内**，你有几天吸过卷烟？

①　0 天

②　1-2 天

③　3-5 天

④　6-9 天

⑤　10-19 天

⑥　20-29 天

⑦　30 天

（　）8. 请回忆一下在**过去的 30 天里**你吸卷烟的那些天。你通常每天吸多少支卷烟？

①　在过去 30 天里我没有吸过卷烟

②　每天少于 1 支

③　每天 1 支

④　每天 2-5 支

⑤　每天 6-10 支

⑥　每天 11-20 支

⑦　每天吸超过 20 支

（　）9. 你通常在哪儿吸烟？**（只能选一个答案）**

①　我不吸烟

②　在家

③　在网吧

④　在学校

⑤　在朋友家

⑥　在社交活动时

⑦　在餐馆

⑧　在其他场所

（　）10. 你是否曾经早晨醒来后就吸烟或者觉得醒来后的第一件事就是想吸烟？

① 我不吸烟

② 不，我没有早晨醒来后就吸烟，也没觉得醒来后的第一件事就是想吸烟

③ 是的，我有时候早晨醒来后就吸烟，或者有时觉得醒来后的第一件事就是想吸烟

④ 是的，我总是早晨醒来后就吸烟，或者总是觉得醒来后的第一件事就是想吸烟

（ ）11. 你想现在戒烟吗？

① 我从不吸烟

② 我现在不吸烟

③ 是

④ 否

（ ）12. 在**过去 12 个月内**，你是否尝试过戒烟？

① 我从不吸烟

② 我过去 12 个月内没有吸过烟

③ 是

④ 否

（ ）13. 你是否接受过帮助你戒烟的帮助或建议（只能选择一个答案）

① 我从不吸烟

② 是的，来自于有组织的戒烟活动或专业人员

③ 是的，来自于朋友

④ 是的，来自于家人

⑤ 是的，来自于有组织的戒烟活动或专业人员，同时也来自朋友或家人

⑥ 没有

（ ）14. 在**过去 7 天内**，当你在家时，有多少天在你家里有人吸烟？

① 0 天

② 1-2 天

③ 3-4 天

④ 5-6 天

⑤ 7 天

（　）15. 在**过去 7 天内，**当你在任何家庭以外的封闭的公共场所时（如教学楼、会场、体育馆、网吧、商店、餐馆、商场、电影院等），有几天有人在那里吸烟？

① 0 天

② 1-2 天

③ 3-4 天

④ 5-6 天

⑤ 7 天

（　）16. 在**过去 7 天内，**当你在任何室外的公共场所时（如操场、人行道、车站、大楼入口、公园等），有几天有人在那里吸烟？

① 0 天

② 1-2 天

③ 3-4 天

④ 5-6 天

⑤ 7 天

（　）17. 在**过去7天内，**当你乘坐公共交通工具时（如火车、公共汽车或者出租车），有几天有人在那里吸烟？

① 在过去 7 天内，我没有乘坐过公共交通工具

② 我乘坐过公共交通工具，但没有人吸烟

③ 1-2 天

④ 3-4 天

⑤ 5-6 天

⑥ 7 天

（　）18. 在过去 30 天内，你是否看到有人在学校的建筑物内或者室外吸烟？

① 是　　② 否

（　）19. 你是否认为别人吸烟产生的烟雾会对你产生危害？

① 肯定不会　② 可能不会　③ 可能会　④ 肯定会

（　）20. 在**过去 30 天内，**是否有人因为你的年龄而拒绝卖给你卷烟？

① 在过去 30 天内，我没有买过卷烟

② 是，有人因为我的年龄而拒绝卖给我卷烟

③ 否，没有人因为我的年龄而拒绝卖给我卷烟

（ ）21. **在过去 30 天内，**你最后一次购买卷烟给自己吸是以怎样的形式购买的?

① 在过去 30 天内，我没有买过卷烟

② 我是按盒买的

③ 我是按支买的

④ 我是按条买的

⑤ 我买了烟丝自己卷的

（ ）22. 在过去 30 天内，你最后一次买来给自己吸的卷烟多少钱一包（每包 20 支）？

① 在过去 30 天内，我没有买过卷烟

② 不到 3 元

③ 3～5 元

④ 6～10 元

⑤ 11～20 元

⑥ 21 元及以上

⑦ 不知道

（ ）23. 在**过去 30 天内，**你是否在电视、广播、互联网、户外广告牌、海报、报纸、杂志或电影上，听到或看到过控烟的信息?

① 是 ②否

（ ）24.在**过去 30 天内，**你是否在电视、录像/视频或者电影中看到有人吸烟?

① 在 过去 30 天内，我没有看过电视、录像/视频或者电影。

② 是

③ 否

（ ）25.在**过去 30 天内，**你是否在烟草零售点看到过烟草产品的广告或者促销（如商店、商场、售货亭等）？

① 在过去 30 天内，我没有去过任何烟草零售点

② 是

③ 否

（ ）26.是否曾经有为烟草公司工作的人给过你免费的烟草产品？

① 是 ② 否

（ ）27. **在过去30天内，**你是否在互联网上看到过烟草产品的广告/视频？

① 在过去30天内，我没用过互联网

② 是

③ 否

（ ）28. 你的父母吸烟吗？

① 两个都不吸

② 两个都吸

③ 只有父亲吸

④ 只有母亲吸

⑤ 不知道

（ ）29. 你的好朋友中是否有人吸烟？

① 没人吸

② 有一些吸

③ 大多数吸

④ 全部吸

（ ）30. 你认为吸烟使年轻人看起来更有吸引力还是相反？

① 更有吸引力

② 减少吸引力

③ 与不吸烟者相比没差别

（ ）31. **在过去12个月内，是否有人在课堂上教过你们**关于烟草使用的后果，如吸烟会导致牙齿发黄、皮肤皱纹或者味道难闻？

① 是 ② 否 ③ 不知道

（ ）32. 在校期间，你一般多久会看到老师在校园内（包括室内和室外区域）吸烟？

① 几乎每天 ② 有时 ③ 从未见过 ④ 不知道

（ ）33.如果你的好朋友给你烟，你会使用它吗？

① 肯定不会 ② 可能不会 ③ 可能会 ④ 肯定会

（ ）34.在**未来的 12 个月内**，你认为自己会使用某种烟草产品吗？

① 肯定不会 ② 可能不会 ③ 可能会 ④ 肯定会

（ ）35.一旦有人已经开始吸烟，你认为会很难戒掉吗？

① 肯定不难 ② 可能不难 ③ 可能难 ④ 肯定难

（ ）36.在庆祝、派对或其他社交聚会的场合，你认为吸烟会让人感到更舒服，还是更不舒服？

① 更舒服

② 更不舒服

③ 吸不吸烟没有差别

（ ）37.你同意还是不同意下面的说法："我认为我可能会喜欢吸卷烟"。

① 我现在吸卷烟

② 非常同意

③ 同意

④ 反对

⑤ 非常反对

（ ）38. 你听说过电子烟吗？

① 是 ② 否

（ ）39.你是否使用过电子烟？（即使只尝试过一、两次也算使用过）

① 是 ② 否

（　）40. 在过去 30 天内，你有几天使用过电子烟?

① 0 天

② 1 - 2 天

③ 3 - 5 天

④ 6 - 9 天

⑤ 10 - 19 天

⑥ 20 - 29 天

⑦ 30 天

（　）41. 请回想一下，你经常使用的电子烟，是否含有尼古丁？

① 我没有使用过电子烟

② 不含尼古丁

③ 含尼古丁

④ 我不知道是否含尼古丁

（　）42. 如果你吸卷烟，也使用电子烟，那么你开始是先吸卷烟的，还是先使用电子烟的？

① 烟 ② 电子烟 ③ 只吸过一种 ④ 两种都不没吸过

（　）43. 如果你的好朋友给你电子烟，你会使用吗？

① 肯定不会 ② 可能不会 ③ 可能会 ④ 肯定会 ⑤ 我不知道

（　）44. 在未来 12 个月，你认为自己有可能使用电子烟吗？

① 肯定不会 ② 可能不会 ③ 可能会 ④ 肯定会 ⑤ 我不知道

（　）45.在过去 30 天内，你是否在下列地方看到过电子烟的广告或者其相关产品的广告（如电子烟烟油）？【可多选】

① 我没有看到过

② 电子烟体验店或者电子烟零售店

③ 商店、超市、便利店、杂货店

④ 报纸杂志

⑤ 电视

⑥ 广播

⑦ 户外广告牌

⑧ 网站（如：淘宝、京东等）

⑨ 网络社交媒体（如：微信、QQ、微博等）

⑩ 体育赛事、文艺演出等社会活动

感谢你参加本次调查！
